# Lyell's syndrome associated with the use of topical agents: case report

**DOI:** 10.17843/rpmesp.2026.433.15468

**Published:** 2026-08-04

**Authors:** Alan P. Murillo-Salas, Estephania B. Díaz-Panuera, Francheska M. Núñez-Gonzales, Aira S. Chávez-Panuera, Fabián L. Cuba-Pacheco, María F. Bernales-Valdivia

**Affiliations:** 1 Hospital Regional Honorio Delgado Espinoza, Arequipa, Peru. Hospital Regional Honorio Delgado Espinoza Arequipa Peru; 2 Universidad Católica de Santa María, Arequipa, Peru. Universidad Católica de Santa María Universidad Católica de Santa María Arequipa Peru

**Keywords:** Stevens-Johnson Syndrome, Anti-Inflammatory Agents, Non-Steroidal, Administration, Topical

## Abstract

Lyell's syndrome (LS) is a severe drug-induced cutaneous adverse reaction, associated with high morbidity and mortality. We present the case of a 41-year-old male who, after the use of topical medications, developed fever, maculopapular lesions, ocular and mucosal involvement, with affectation of more than 30% of the body surface area. Cutaneous biopsy ruled out other pathologies. Management included systemic methylprednisolone, aluminum subacetate, topical mupirocin, ophthalmic prednisolone, and ophthalmic hypromellose, with a favorable outcome and without complications. This case highlights the importance of considering topical medications as possible triggers for LS, as well as the need for early diagnosis and timely management. Early identification and a multidisciplinary therapeutic approach are fundamental to improve clinical outcomes and reduce the morbidity and mortality associated with this entity.

## INTRODUCTION

Lyell's syndrome (LS), also known as toxic epidermal necrolysis (TEN), is a severe skin condition similar to a burn. It compromises not only the skin but also the mucous membranes and the epithelium of various organs. It is characterized by a dark morbilliform rash that rapidly progresses to vesicles, blisters, and skin detachment. In approximately 90%, it originates as an adverse drug reaction ^(^^1^^,^^2^.

LS is considered a continuation of Stevens-Johnson syndrome (SJS), differing only by the percentage of affected body surface area (BSA) ^(^^4^. SJS is characterized by involvement of less than 10% of the BSA, while LS by epidermal detachment equal to or greater than 30% of the BSA. An overlap form may occur between both when the involvement is between 10% and 30% of BSA ^3^. Therefore, LS is associated with a higher mortality rate, reaching 50% compared to SJS at 10% ^(^^4^.

In most cases of SL, the trigger corresponds to an adverse reaction to a systemically administered drug, such as antibiotics ^(^^4^. However, severe cutaneous reactions caused by topically used drugs are rare, and there are few reports associated with the use of non-steroidal anti-inflammatory drugs (NSAIDs) such as methyl salicylate. Systemic toxicity from topical salicylates such as methyl salicylate has been described, which evidences its capacity for cutaneous absorption ^(^^4^; however, no reports are available that link it to TEN.

Therefore, the case of a patient who developed LS after the application of topical creams is described. Based on this clinical case, it is important to consider that topical medications can also act as possible triggers for serious skin conditions.

## CASE REPORT

A 41-year-old mestizo male patient, native and from Arequipa, and a glassmaker by occupation. He was admitted to the internal medicine service in October 2024. He presented with fever, cutaneous burning, and a rosy spot on his forehead that progressively extended to his face, trunk, and extremities for 10 days.

Initially, he was hospitalized for 6 days in a private clinic where he received treatment and a skin biopsy was performed; however, he requested voluntary discharge and subsequently went to the hospital presenting with a severe burn-like appearance, blurred vision, fever, and generalized pain. For 5 months, he had presented with mechanical-type pain in the cervical and lumbar spine, both elbows, and knees, for which topical agents were applied daily. For the first 3 months, 50-gram ointments containing methyl salicylate, menthol, eucalyptus oil, camphor, arnica oil, and molle oil, known as «snake tallow», were applied two to three times a day upon waking, in the afternoon, and before going to bed. Subsequently, for the next 2 months, the pain persisted and he increased the application doses every four to six hours, especially before and after working. He denied suffering from allergies, diseases, or medication intake (NSAIDs, antibiotics, corticosteroids, etc.) by any other route of administration. The presence of the topical agent was evaluated using the Naranjo algorithm, which indicated a category of 6 points (table 1).

**Table 1:**
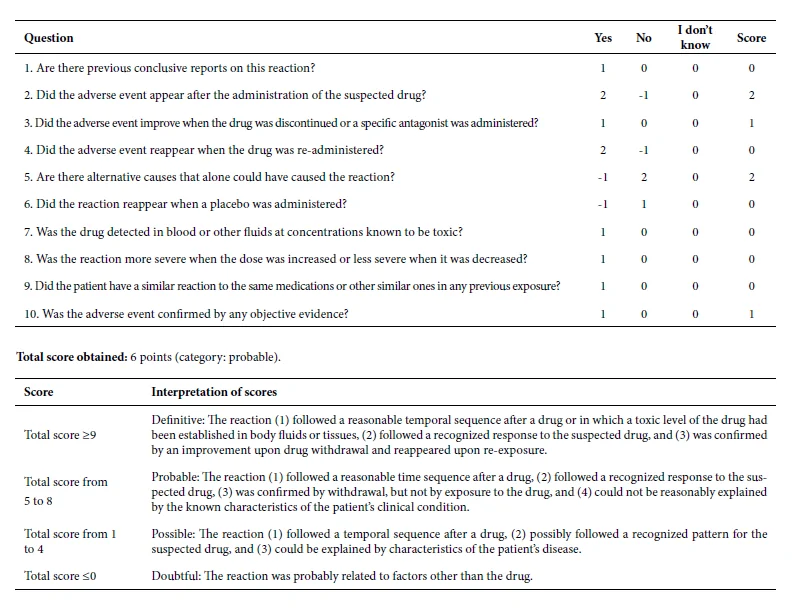
Naranjo Algorithm: probability scale for adverse drug reactions.

On physical examination, she presented with a temperature of 38.2 °C. The dermatological examination evidenced facial involvement in perioral regions, lips, and oral mucosa with necrotic skin and hemorrhagic crusts of central facial distribution with denuded skin covering eyelids, nose, and mouth, hindering oral opening. Additionally, violaceous maculopapular erythema with multiple blisters, painful on palpation, was observed, disseminated in cervical, supraclavicular, thoracic, abdominal, and extremity regions, with involvement greater than 30% of BSA. Likewise, she presented with oral, ocular, and genital mucosal involvement, accompanied by purulent discharge and a positive Nikolsky sign (figure 1). No lymphadenopathies were palpable. The ophthalmological examination evidenced absence of eyelashes on the palpebral margin with limitation for eyelid opening and closing, in addition to hyperemic conjunctivae in both eyes. The rest of the physical examination was normal.

**Figure 1:**
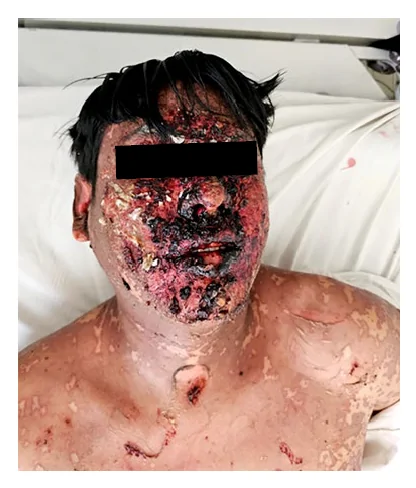
Face involvement in perioral, labial, and oral mucosa regions with necrotic skin and areas with hemorrhagic crusts of centrofacial distribution with denuded skin covering eyelids, nose, and mouth; additionally violaceous maculopapular erythema with multiple flaccid bullae in cervical, supraclavicular, and thoracic regions (photo taken with patient's consent).

Laboratory tests recorded hemoglobin of 17.3 g/dL, leukocytes at 9950 /mm^3^ and platelets at 172,000 /mm^3^ with elevated C-reactive protein (CRP) 37.6 mg/dL. Within biochemistry, creatinine was recorded as 1.18 mg/dL, alanine aminotransferase units of 37 U/L and alkaline phosphatase of 102 U/L, gamma-glutamyl transpeptidase of 50 U/L, albumin of 4.5 g/dL, and electrolytes such as sodium 136 mEq/L, chloride 107 mEq/L, and potassium 4.49 mEq/L (Table 2).

**Table 2:** Initial Laboratory Tests.

Parameter		Result
Blood count		
	Hemoglobin	17.3 g/dL
	Leukocytes	9950 /mm³
	Neutrophils	80%
	Lymphocytes	9%
	Monocytes	9%
	Eosinophils	2%
	Basophils	2%
	Platelets	172 000 /mm³
Biochemistry and electrolytes		
	Glucose	112.7 mg/dL
	Creatinine	1.18 mg/dL
	Total Bilirubin	1.4 mg/dL
	Direct bilirubin	0.5 mg/dL
	Indirect bilirubin	0.9 mg/dL
	Aspartate aminotransferase	30.8 U/L
	Alanine aminotransferase	37.3 U/L
	Alkaline phosphatase	102 IU/L
	Gamma-glutamyl transpeptidase	50 U/L
	Total proteins	7.8 g/dL
	Albumin	4.5 g/dL
	Globulin	3.3 g/dL
	Sodium	136 mEq/L
	Chlorine	107 mEq/L
	Potassium	4.49 mEq/L
Immunology and serology		
	C-reactive protein	37.6 mg/dL
	HIV-1/HIV-2	Non-reactive

The fundus examination showed slightly opaque corneas compatible with exposure keratitis. Skin biopsy showed subepidermal blisters with extravasation of red blood cells, dermis with perivascular lymphocytic infiltrate, and necrotic keratinocytes (figure 2).

**Figure 2:**
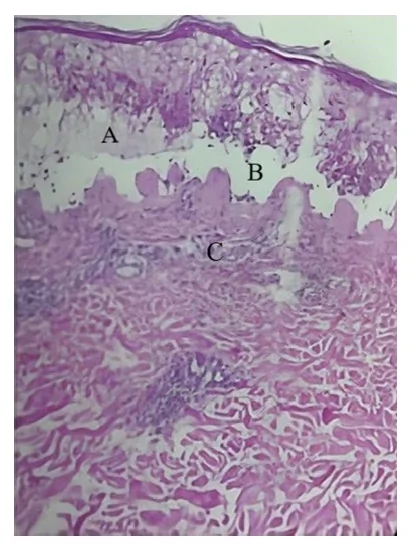
Histopathological slide, hematoxylin-eosin stain, 10x magnification, showing necrotic keratinocytes (A), subepidermal blisters (B) and dermal infiltrate (C).

The patient received multidisciplinary management for severe burns, with fluid support with crystalloids such as Ringer's Lactate 1000 mL at a dose of three bottles per day with subsequent reduction of hydration due to the patient's better oral tolerance with a high-protein diet at a dose of 1.2 g/kg in addition to trace elements. Pharmacological treatment with methylprednisolone 1 gram intravenous every 24 hours and chlorphenamine 10 milligrams intravenous every 8 hours, both for 3 days, subsequently received oral prednisone at a dose of 1 mg/kg with progressive weaning every five days.

Additionally, to prevent infections, topical treatment was received with aluminum subacetate solution one application every 24 hours, mupirocin 2% cream every 8 hours. Corneal damage was treated with ophthalmic prednisolone 1 drop in each eye every six hours and ophthalmic hypromellose 1 drop in each eye every 4 hours.

All topical medication was administered for 30 days where close observation and follow-up of the patient were maintained, the dermatological examination evidenced improvement in mouth opening, which facilitated better oral intake and swallowing, with less compromise of necrosis and hemorrhage in perioral regions, lips, and oral mucosa (figure 3). The maculopapular erythema persisted, but without bullae and with control of local pain and applications of topical creams to prevent opportunistic infections, control scars and hyperpigmented lesions ^(^^4^.

**Figure 3:**
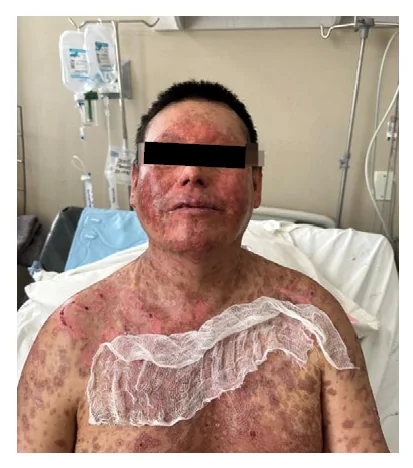
Skin with favorable evolution; absence of necrotic scabs and improved skin integrity are observed (photo taken with patient's consent).

The ophthalmological examination showed palpebral improvement, and also progressive resolution of bilateral conjunctivitis, without evidence of corneal epithelial or pseudomembranous detachment. Treatment was maintained to prevent complications such as opportunistic infections and visual loss, frequent in LS ^4^.

## DISCUSSION

LS is a severe skin reaction caused by extensive necrosis of skin and mucous membranes ^4^, its management usually requires a specialized unit ^5^, due to its similarity with severely burned patients. The pathophysiology of LS involves a type IV hypersensitivity reaction mediated by T cells that produces epidermal necrosis, with a characteristic histopathological finding of necrotic keratinocytes ^5^^,^^6^.

LS is usually associated with antiepileptics, antivirals, NSAIDs, allopurinol, and antibiotics; it can also be related to infections, neoplasms, connective tissue disorders, or graft-versus-host disease ^7^^,^^8^. Most cases (80-85%) correspond to idiosyncratic drug reactions. Its incidence is 1-2 cases per million people, with a mortality of 25-35% that can reach 75% without adequate management. It most frequently affects women (2-3:1) and extreme ages (<5 or >64 years). Survivors may present with skin scars or ocular damage ^6^^,^^9^.

Salicylates are derivatives of salicylic acid with analgesic and anti-inflammatory properties that act primarily through the inhibition of cyclooxygenase (COX), reducing the synthesis of prostaglandins involved in inflammation and pain. Methyl salicylate is a lipophilic compound capable of penetrating the stratum corneum and being hydrolyzed in the skin to active salicylic acid. This metabolite exerts local anti-inflammatory effects, produces vasodilation, and acts as a rubefacient by increasing blood flow in the applied area. However, it can be absorbed systemically and cause toxicity by altering cellular metabolism ^10^^,^^11^^,^^12^.

Although TEN is associated with the use of systemic drugs, some topical medications can also produce significant adverse effects due to their cutaneous absorption. A case of systemic toxicity following the application of a methyl salicylate cream has been reported, demonstrating that these compounds can generate relevant clinical complications ^10^. Likewise, severe cutaneous reactions associated with topical medications have been described, such as a case of SJS triggered by ophthalmic ofloxacin ^13^, while Noiles and Pratt documented allergic contact dermatitis from Vicks VapoRub, confirmed by positive patch tests to eucalyptus and cedar oil, which demonstrates the sensitizing potential of complex topical preparations ^14^. This demonstrates that even topical formulations of different pharmacological classes can induce systemic adverse reactions.

The patient was a 41-year-old male, outside the typical epidemiological pattern, with a reaction induced by a topical NSAID. The diagnosis was LS; although, it must be differentiated from other severe cutaneous adverse reactions (SCAR), such as SJS, drug reaction with eosinophilia and systemic symptoms (DRESS) syndrome, and acute generalized exanthematous pustulosis (AGEP). SJS and LS are part of the same spectrum without clear boundaries. Their diagnosis is clinical because they present erythematous macules, atypical targets, and mucosal erosions due to necrosis; however, they differ by the percentage of affected BSA: BSA <10% is SJS, 10-30% is an overlap between SJS/LS, and >30% is LS ^15^^-^^18^. Biopsy helps rule out other pathologies but does not distinguish between the two; histopathological findings are characterized by presenting necrotic keratinocytes, subepidermal bullae, and perivascular lymphocytic infiltrate ^(^^16^.

DRESS appears 2-6 weeks after drug exposure, with acrofacial lesions ^5^ and facial edema, especially in the head and neck ^6^. DRESS is also characterized by hematological alterations, fever, lymphadenopathies, and hepatic and renal involvement. Histopathologically, it differs by the absence of full-thickness necrosis. Treatment in both includes drug discontinuation, using systemic corticosteroids, such as prednisolone ^5^^,^^6^. The patient did not meet DRESS criteria due to the lower severity of systemic symptoms.

AGEP appears 24-48 hours after drug exposure and manifests with sterile non-follicular pustules on a diffuse erythematous background, initially in intertriginous areas or the face, and then on the trunk and extremities ^18^. Pustule coalescence can simulate SL as there is a positive pseudo-Nikolsky sign ^5^^,^^18^. Histologically, it shows subcorneal spongiform pustules, edema in the papillary dermis, and perivascular neutrophil infiltrate. Corticosteroids can shorten hospitalization, and improvement usually occurs 2 weeks after drug withdrawal ^5^^,^^6^. In this case, violaceous maculopapular erythema with denuded skin characteristic of LS was observed.

Hypereosinophilic syndrome (HES) is a rare disease characterized by persistent eosinophilia (>1500/μL) and multisystemic involvement ^19^. It does not cause epidermal necrosis, although it can generate thrombosis and cardiac dysfunction ^20^. Treatment includes corticosteroids, immunosuppressants, or mepolizumab in refractory cases ^20^. In this case, HES was ruled out due to absence of eosinophilia and organ damage.

The use of corticosteroids in LS is controversial and contradictory; although their prolonged use increases the risk of infections, in early stages it can improve survival and prevent cicatricial sequelae ^7^^,^^8^. The patient received short-term intravenous methylprednisolone 1g and continued with a progressive prednisone weaning to reduce initial inflammation and improve survival ^(^^7^. However, other studies reveal lower mortality in patients who received a lower dose of corticosteroids and combined use with cyclosporine for periods of 5 to 7 days. In addition, the prevention of infections and ocular sequelae was necessary, thus, the use of topical treatment with aluminum subacetate, mupirocin, and ophthalmic treatment was rapidly indicated ^7^^,^^8^.

In conclusion, a TEN associated with methyl salicylate is described. This evidences that even topical products can trigger severe cutaneous reactions. Early suspension of the suspected agent improves the prognosis and reduces morbidity and dermatological sequelae. Likewise, it should be considered that ointments known as «sebo de culebra» are not regulated medicinal products and their use may represent a health risk.
