# Supplementary material for: Lyell's syndrome associated with the use of topical agents: case report
Source: Rev Peru Med Exp Salud Publica. 2026 Aug 4;43(3):402–7. doi: 10.17843/rpmesp.2026.433.15468 (PMC13718553; doi:10.17843/rpmesp.2026.433.15468)
Supplement: Translation

# Translation: Síndrome de Lyell asociado al uso de agentes de aplicación tópica: reporte de caso

**Authors:** Alan P. Murillo-Salas, Estephania B. Díaz-Panuera, Francheska M. Núñez-Gonzales, Aira S. Chávez-Panuera, Fabián L. Cuba-Pacheco, María F. Bernales-Valdivia

**Affiliations:** 1 Hospital Regional Honorio Delgado Espinoza, Arequipa, Perú.; 2 Universidad Católica de Santa María, Arequipa, Perú.

**Keywords:** Síndrome de Stevens Johnson, Antiinflamatorios no Esteroideos, Administración Tópica

## Abstract

El síndrome de Lyell (SL) es una reacción adversa cutánea grave inducida por fármacos, asociada a alta morbilidad y mortalidad. Se presenta el caso de un varón de 41 años que, tras el uso de medicamentos tópicos, desarrolló fiebre, lesiones maculopapulares, compromiso ocular y mucoso, con afectación de más del 30% del área de superficie corporal. La biopsia cutánea descartó otras patologías. El manejo incluyó metilprednisolona sistémica, subacetato de aluminio, mupirocina tópica, prednisolona oftálmica e hipromelosa oftálmica, con evolución favorable y sin complicaciones. Este caso resalta la importancia de considerar a los medicamentos tópicos como posibles desencadenantes de SL así como la necesidad de un diagnóstico precoz y manejo oportuno. La identificación temprana y un enfoque terapéutico multidisciplinario son fundamentales para mejorar los resultados clínicos y reducir la morbimortalidad asociadas a esta entidad.

## INTRODUCCIÓN

El síndrome de Lyell (SL) también denominado necrólisis epidérmica tóxica (NET) es una afección cutánea grave similar a una quemadura. Compromete no solo la piel sino también las mucosas y el epitelio de diversos órganos. Se caracteriza por una erupción morbiliforme oscura que progresa rápidamente a vesículas, ampollas y desprendimiento cutáneo. En aproximadamente el 90% se origina como una reacción adversa a medicamentos ^(^^1^^,^^2^.

El SL es considerada una continuación del síndrome de Stevens-Johnson (SSJ) diferenciándose únicamente por el porcentaje de área de superficie corporal (ASC) afectada ^(^^4^. El SJS se caracteriza por comprometer menos del 10% del ASC, mientras que el SL por un desprendimiento epidérmico igual o mayor al 30% del ASC. Entre ambos puede presentarse una forma de superposición cuando la afectación se encuentra entre el 10% y el 30% de AC ^3^, por ello el SL se asocia a una tasa de mortalidad más alta alcanzando un 50% a comparación de SSJ en un 10% ^(^^4^.

En la mayoría de los casos de SL el desencadenante corresponde a una reacción adversa a un fármaco de administración sistémica, como antibióticos ^(^^4^. Sin embargo, las reacciones cutáneas graves provocadas por medicamentos de uso tópico son poco frecuentes y existen escasos reportes asociados al uso de antiinflamatorios no esteroideos (AINES) como salicilato de metilo. Se ha descrito toxicidad sistémica por salicilatos tópicos como salicilato de metilo lo que evidencia su capacidad de absorción cutánea ^(^^4^; no obstante, no se disponen de reportes que lo relacionen a NET.

Por ello, se describe el caso de un paciente que desarrolló SL tras la aplicación de cremas tópicas. A partir de este caso clínico, es importante considerar que los medicamentos tópicos también pueden actuar como posibles desencadenantes de afecciones cutáneas graves.

## REPORTE DE CASO

Un paciente varón mestizo de 41 años, natural, procedente de Arequipa y de ocupación vidriero. Fue admitido al servicio de medicina interna en octubre del año 2024. Presentó alza térmica, ardor cutáneo y una mancha rosácea en la frente que se extendió progresivamente al rostro, tronco y extremidades durante 10 días.

Inicialmente fue hospitalizado por 6 días en clínica particular donde recibió tratamiento y se realizó biopsia cutánea; sin embargo, solicitó su retiro voluntario y posteriormente acudió al hospital por presentar aspecto de gran quemado, visión borrosa, fiebre y dolor generalizado. Desde hacía 5 meses presentó dolor tipo mecánico en columna cervical, lumbar, ambos codos y rodillas por lo que se aplicó diariamente agentes tópicos. Los primeros 3 meses se aplicó ungüentos de 50 gramos que contenían salicilato de metilo, mentol, aceite de eucalipto, alcanfor, aceite de árnica y aceite de molle conocida como «sebo de culebra» entre dos a tres veces al día al levantarse, por la tarde y antes de acostarse. Posteriormente, los siguientes 2 meses persistió con el dolor y aumentó las dosis de aplicación cada cuatro a seis horas sobre todo antes y después de laborar. Negó padecer alergias, enfermedades e ingesta de medicamentos (AINES, antibióticos, corticoides, etc.) por cualquier otra vía de administración. Se evaluó la presencia del agente tópico usando el algoritmo de Naranjo el cual indicó una categoría de 6 puntos (tabla 1).

**Tabla 1:** Algoritmo de Naranjo: escala de probabilidad de reacciones adversas a medicamentos.

En el examen físico presentó temperatura 38,2 °C. El examen dermatológico evidenció compromiso de rostro en regiones perioral, labios y mucosa oral con piel necrosada y costras hemorrágicas de distribución centro facial con piel denudada que cubría párpados, nariz y boca dificultando la apertura bucal. Se observó además eritema maculopapular violáceo con múltiples flictenas, dolorosas a la palpación, diseminadas en regiones cervical, supraclavicular, torácica, abdominal y extremidades, con compromiso mayor al 30% de ASC. Asimismo, presentó afectación de mucosas oral, ocular y genital, acompañada de secreción purulenta y signo de Nikolsky positivo (figura 1). No se palparon linfadenopatías. El examen oftalmológico evidenció ausencia de pestañas en el borde palpebral con limitación para la apertura y cierre palpebral, además de conjuntivas hiperémicas en ambos ojos. El resto del examen físico fue normal.

**Figura 1:** Compromiso de rostro en regiones perioral, labios y mucosa oral con piel necrosada y áreas con costras hemorrágicas de distribución centro facial con piel denudada que cubren párpados, nariz y boca; además eritema violáceo maculopapular con múltiples flictenas en regiones cervical, supraclavicular y torácica (foto tomada con consentimiento del paciente).

Los exámenes de laboratorio registraron hemoglobina de 17,3 g/dL, leucocitos en 9950 /mm^3^ y plaquetas en 172 000 /mm^3^ con elevación de proteína c reactiva (PCR) 37,6 mg/dL. Dentro de la bioquímica se registró creatinina 1,18 mg/dL, unidades alanina aminotransferasa de 37 u/L y fosfatasa alcalina de 102 u/L, gamma-glutamil transpeptidasa de 50 U/L, albumina de 4,5 g/dL y electrolitos como sodio 136 mEq/L, cloro 107 mEq/L y potasio 4,49 mEq/L (tabla 2).

**Tabla 2:** Exámenes de laboratorio iniciales.

Parámetro		Resultado
Hemograma		
	Hemoglobina	17,3 g/dL
	Leucocitos	9950 /mm³
	Neutrófilos	80%
	Linfocitos	9%
	Monocitos	9%
	Eosinófilos	2%
	Basófilos	2%
	Plaquetas	172 000 /mm³
Bioquímica y electrolitos		
	Glucosa	112,7 mg/dL
	Creatinina	1,18 mg/dL
	Bilirrubinas totales	1,4 mg/dL
	Bilirrubina directa	0,5 mg/dL
	Bilirrubina indirecta	0,9 mg/dL
	Aspartato aminotransferasa	30,8 U/L
	Alanina aminotransferasa	37,3 U/L
	Fosfatasa alcalina	102 UI/L
	Gamma-glutamil transpeptidasa	50 U/L
	Proteínas totales	7,8 g/dL
	Albúmina	4,5 g/dL
	Globulina	3,3 g/dL
	Sodio	136 mEq/L
	Cloro	107 mEq/L
	Potasio	4,49 mEq/L
Inmunología y serología		
	Proteína C reactiva	37,6 mg/dL
	VIH-1/VIH-2	No reactivo

El fondo de ojo evidenció córneas levemente opacas compatibles con queratitis por exposición. La biopsia cutánea evidenció ampollas subepidérmicas con extravasación de hematíes, dermis con infiltrado linfocitario perivascular y queratinocitos necróticos (figura 2).

**Figura 2:** Lámina histopatológica, tinción hematoxilina-eosina, aumento 10x, donde se observa queratinocitos necróticos (A), ampollas subepidérmicas (B) e infiltrado dérmico (C).

El paciente recibió manejo multidisciplinario tipo gran quemado, con soporte hídrico con cristaloides como Lactato Ringer 1000 mL a dosis de tres frascos al día con posterior disminución de hidratación por mejor tolerancia oral del paciente con dieta alta en proteínas a dosis de 1.2 g/kg además de oligoelementos. El tratamiento farmacológico con metilprednisolona 1 gramo endovenoso cada 24 horas y clorfenamina 10 miligramos endovenosos cada 8 horas ambos por 3 días, posteriormente recibió prednisona vía oral a dosis de 1 mg/kg con progresivo destete cada cinco días.

Además, para prevenir infecciones recibió tratamiento tópico con solución de subacetato de aluminio una aplicación cada 24 horas, mupirocina 2% crema cada 8 horas. Se trato el daño corneal con prednisolona oftálmica 1 gota en cada ojo cada seis horas e hipromelosa oftálmica 1 gota en cada ojo cada 4 horas.

Toda la medicación tópica se administró durante 30 días donde se mantuvo observación y seguimiento minucioso del paciente, el examen dermatológico evidenció mejoría de la apertura bucal que facilito la mejor ingesta oral y deglución, con menor compromiso de necrosis y hemorragia en regiones perioral, labios y mucosa oral (figura 3). El eritema maculopapular se mantenía, pero sin flictenas y con control del dolor local y aplicaciones de cremas tópicas para prevenir infecciones oportunistas, control de cicatrices y lesiones hiperpigmentadas ^(^^4^.

**Figura 3:** Piel con evolución favorable; se observa ausencia de costras necróticas y mejoría de la integridad cutánea (foto tomada con consentimiento del paciente).

El examen oftalmológico mostro mejoría palpebral, además y resolución progresiva de la conjuntivitis bilateral, sin evidencia de desprendimiento epitelial corneal, ni pseudomembranosa, se mantuvo tratamiento para prevenir complicaciones como infecciones oportunistas y pérdida visual, frecuentes en el SL ^4^.

## DISCUSIÓN

El SL es una reacción cutánea grave causada por una necrosis extensa de piel y mucosas ^4^, su manejo suele requerir una unidad especializada ^5^, debido a su similitud con el paciente gran quemado. La fisiopatología del SL implica una reacción de hipersensibilidad tipo IV mediada por células T que produce necrosis epidérmica, con hallazgo histopatológico característico de queratinocitos necróticos ^5^^,^^6^. 

El SL suele asociarse a antiepilépticos, antivirales, AINES, alopurinol y antibióticos también puede relacionarse con infecciones, neoplasias, trastornos del tejido conectivo o enfermedad de injerto contra huésped ^7^^,^^8^. La mayoría de los casos (80-85%) corresponde a reacciones idiosincrásicas a fármacos. Su incidencia es de 1-2 casos por millón de personas, con una mortalidad del 25-35% que puede llegar 75% sin manejo adecuado. Afecta con mayor frecuencia a mujeres (2-3:1) y edades extremas (<5 o >64 años). Los sobrevivientes pueden presentar cicatrices cutáneas o daño ocular ^6^^,^^9^. 

Los salicilatos son derivados del ácido salicílico con propiedades analgésicas y antiinflamatorios que actúan principalmente mediante la inhibición de la ciclooxigenasa (COX) reduciendo la síntesis de prostaglandinas implicadas en la inflamación y el dolor. El salicilato de metilo, es un compuesto lipofílico capaz de penetrar el estrato córneo y ser hidrolizado en la piel a ácido salicílico activo. Este metabolito ejerce efectos antiinflamatorios locales, produce vasodilatación y actúa de rubefaciente al aumentar el flujo sanguíneo en la zona aplicada. Sin embargo, puede absorberse sistemáticamente y causar toxicidad al alterar el metabolismo celular ^10^^,^^11^^,^^12^.

Aunque la NET se asocia al uso de fármacos sistémicos, algunos medicamentos tópicos también pueden producir efectos adversos significativos debido a su absorción cutánea. Se ha reportado un caso de toxicidad sistémica tras la aplicación de una crema con salicilato de metilo, lo que demuestra que estos compuestos pueden generar complicaciones clínicas relevantes ^10^. Asimismo, se han descrito reacciones cutáneas graves asociadas a medicamentos tópicos, como un caso de SJS desencadenado por ofloxacino oftálmico ^13^, mientras que Noiles y Pratt documentaron dermatitis alérgica de contacto por Vicks VapoRub, confirmada mediante pruebas epicutáneas positivas a aceite de eucalipto y cedro, lo que demuestra el potencial sensibilizante de preparados tópicos complejos ^14^. Esto evidencia que incluso las formulaciones tópicas de diferentes clases farmacológicas pueden inducir reacciones adversas sistémicas.

El paciente fue un varón de 41 años, fuera del patrón epidemiológico típico, con una reacción inducida por un AINE tópico. El diagnóstico fue SL; aunque, debe diferenciarse de otras reacciones adversas cutáneas graves (SCAR), como el SSJ, el síndrome de hipersensibilidad a fármacos con eosinofilia y síntomas sistémicos (DRESS) y la pustulosis exantemática generalizada aguda (PEGA). El SSJ y SL forman parte del mismo espectro sin límites claros. Su diagnóstico es clínico porque presentan máculas eritematosas, dianas atípicas y erosiones mucosas por necrosis, sin embargo, se diferencian por el porcentaje de ASC afectada: ASC <10% es SJS, 10-30% es una superposición entre SJS/SL y >30% es SL ^15^^-^^18^. La biopsia ayuda a descartar otras patologías, pero no distingue entre ambas, los hallazgos histopatológicos se caracterizan por presentar queratinocitos necróticos, ampollas subdérmicas e infiltrado linfocítico perivascular ^16^.

El DRESS aparece 2-6 semanas después de la exposición al fármaco, con lesiones acrofaciales ^5^ y edema facial, especialmente en cabeza y cuello ^6^. El DRESS se caracteriza además por alteraciones hematológicas, fiebre, linfadenopatías y compromiso hepático y renal. Histopatológicamente se diferencia por la ausencia de necrosis de espesor total. El tratamiento en ambos incluye suspensión del fármaco, usar corticoides sistémicos, como prednisolona ^5^^,^^6^. El paciente no cumplió criterios de DRESS por la menor gravedad de síntomas sistémicos.

La PEGA aparece 24-48 horas tras la exposición al fármaco y se manifiesta con pústulas estériles no foliculares sobre fondo eritematoso difuso, inicialmente en áreas intertriginosas o cara y luego en tronco y extremidades ^18^. La coalescencia de pústulas puede simular SL al haber un pseudo-Nikolsky positivo ^5^^,^^18^. Histológicamente muestra pústulas espongiformes subcorneales, edema en dermis papilar e infiltrado perivascular de neutrófilos. Los corticosteroides pueden acortar la hospitalización y suele haber mejoría a 2 semanas del retiro del fármaco ^5^^,^^6^. En este caso se observó eritema maculopapular violáceo con piel denudada característico de SL.

El síndrome hipereosinofílico (SHE) es una enfermedad rara caracterizada por eosinofilia persistente(>1500/μL) y compromiso multisistémico ^19^. No produce necrosis epidérmica, aunque puede generar trombosis y disfunción cardiaca ^20^. El tratamiento incluye corticosteroides, inmunosupresores o mepolizumab en casos refractarios ^20^. En este caso se descartó SHE debido a ausencia de eosinofilia y daño orgánico.

El uso de corticosteroides en SL es controvertido y contradictorio, aunque su uso prolongado aumenta el riesgo de infecciones, en fases tempranas puede mejorar la supervivencia y prevenir secuelas cicatriciales ^7^^,^^8^. El paciente recibió metilprednisolona 1g endovenoso a corto plazo y continuo con destete progresivo de prednisona para reducir la inflamación inicial y mejorar la supervivencia ^(^^7^. Sin embargo, otros estudios revelan menor mortalidad en pacientes que recibieron menor dosis de corticoesteroides y uso de combinado con ciclosporina en periodos de 5 a 7 días. Además, fue necesaria la prevención de infecciones y secuelas oculares por lo que se indicó rápidamente el uso de tratamiento tópico con subacetato de aluminio, mupirocina y tratamiento oftálmico ^7^^,^^8^.

En conclusión, se describe una NET asociada a salicilato de metilo. Esto evidencia que incluso productos tópicos pueden desencadenar reacciones cutáneas graves. La suspensión precoz del agente sospechoso mejora el pronóstico y reduce la morbilidad y secuelas dermatológicas. Asimismo, debe considerarse que las pomadas conocidas como «sebo de culebra» no son productos medicinales regulados y su uso puede representar un riesgo para la salud.
